# Quantum interference effects at room temperature in OPV-based single-molecule junctions

**DOI:** 10.1186/1556-276X-8-234

**Published:** 2013-05-16

**Authors:** Carlos R Arroyo, Riccardo Frisenda, Kasper Moth-Poulsen, Johannes S Seldenthuis, Thomas Bjørnholm, Herre SJ van der Zant

**Affiliations:** 1 Kavli Institute of Nanoscience, Delft University of Technology, Lorentzweg 1, Delft, 2628 CJ, The Netherlands; 2 Nano-Science Center, Department of Chemistry, University of Copenhagen, Universitetsparken 5, Copenhagen, 2100, Denmark; 3 Department of Chemical and Biological Engineering, Chalmers University of Technology, Gothenburg, 41296, Sweden

**Keywords:** Single-molecule transport, Quantum interference, Break junctions, Non-equilibrium Green’s functions

## Abstract

Interference effects on charge transport through an individual molecule can lead to a notable modulation and suppression on its conductance. In this letter, we report the observation of quantum interference effects occurring at room temperature in single-molecule junctions based on oligo(3)-phenylenevinylene (OPV3) derivatives, in which the central benzene ring is coupled to either *para*- or *meta*-positions. Using the break-junction technique, we find that the conductance for a single *meta*-OPV3 molecule wired between gold electrodes is one order of magnitude smaller than that of a *para*-OPV3 molecule. Theoretical calculations confirm the occurrence of constructive and destructive interference in the *para*- and *meta*-OPV3 molecules respectively, which arises from the phase difference of the transmission coefficients through the molecular orbitals.

## Background

Fundamental research regarding the quantum transport mechanisms in individual molecules is of vital importance for molecular electronics. In the realization of a metal-molecule-metal junction, the Fermi energy of the metal lies within a relatively large HOMO-LUMO gap (HOMO, highest occupied molecular orbital; LUMO, lowest unoccupied molecular orbital) and the electrons tunnel coherently across the molecular junction. In this description, the conductance of a single-molecule decays exponentially as a function of its length, and this has been indeed confirmed for prototypical molecular backbones like non-conjugated alkane chains [1,2] and π-conjugated molecular wires [3,4]. However, such a simple tunneling picture does not take into account the effect of quantum interference that can strongly influence charge transport at the molecular scale [5,6]. The understanding and control of quantum interference phenomena at the molecular scale may lead to single-molecule devices with new functionalities and, therefore, are a subject of increased scientific interest both theoretically [7-11] and experimentally [12-16]. An archetypal system, in which quantum interference effects are expected, is a single benzene ring [5]. It has been shown theoretically that a benzene ring connected between two electrodes in a *para* configuration should have a conductance that is several orders of magnitude higher than that of a *meta* configuration [7,17]. This reduction in the molecular conductance can be understood in terms of interference effects occurring between electron waves propagating through different pathways. These pathways are separated in energy, and the interference between their transmission components can lead to constructive or destructive interference [7,8,18,19].

Over the years, a large variety of techniques and methods have been employed to investigate the electronic properties of individual molecules connected between metallic electrodes. In particular, the advances obtained during the last decade using the break-junction technique [1] have revolutionized our understanding about charge transport through single-molecule junctions. This technique consists in repeatedly moving two metallic electrodes into and out of contact with each other in the presence of molecules equipped with suitable anchoring groups. During the separation of the electrodes, signatures of the formation of molecular junctions can be observed and statistical analysis permits to obtain the most probable conductance values for a single-molecule junction. From these studies, we now largely understand how the anchoring groups [20-22], the length [1,2], the conformation [23], and the conjugation [3] of the molecule influence its conductance. These achievements together with the progress in computational methods [24] have stimulated molecular designs with new functionalities.

In the present study, the effect of quantum interference on electron transport through a single benzene ring is explored by considering two specifically designed oligo(3)-phenylenevinylene (OPV3) derivatives in which the central benzene ring is coupled either in a *para* or *meta* configuration. Details concerning the synthetic procedure for the *para*-OPV3 have been previously reported [25] while for the *meta*-OPV3 are given in the Additional file 1. The low-bias conductance of single-molecule junctions bonded via thiol groups to gold electrodes is measured and statistically analyzed using the mechanically controlled break-junction (MCBJ) technique and conductance histograms. In a recent work [26], we reported signatures of quantum interference effects through a benzene ring coupled to thienyl anchoring groups by ethynyl spacers. The observation of interference effects in both systems indicates that the coupling to the central benzene ring determines the occurrence of quantum interference effects, while the spacers and anchoring groups slightly tune the conductance through the molecular junction.

## Methods

**Figure 1:**
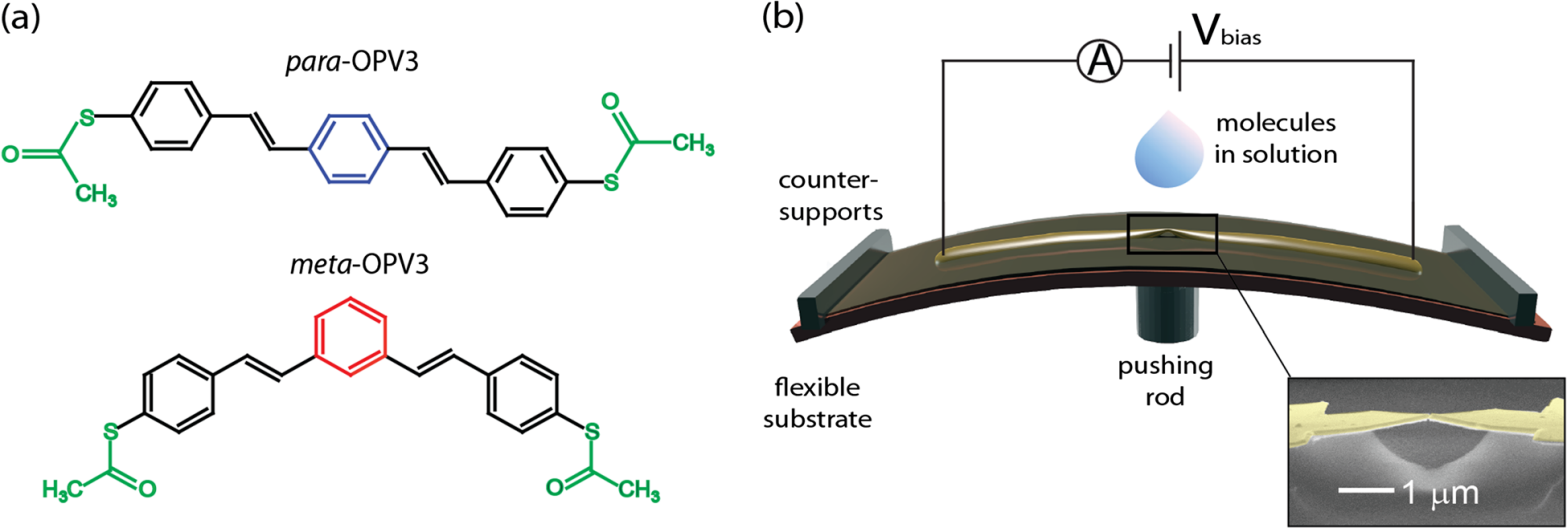
**Structures of OPV3-based molecules and MCBJ setup.** (**a**) Structures of OPV3-based molecules studied in this work. The *para*- (blue) and *meta*- (red) coupled benzene rings are connected to acetyl-protected thiols (green) by vinyl spacers (black). (**b**) Scheme of the mechanically controlled break-junction (MCBJ) setup. Inset, false-color scanning electron micrograph of a MCBJ device.

We explore quantum interference effects in charge transport through a single benzene ring by measuring the low-bias conductance of two different OPV3 molecules depicted in Figure 1a. The molecules consist of a single benzene ring coupled in a *para* or *meta* configuration to vinyl spacers and terminated by acetyl-protected thiol anchoring groups. The vinyl spacers provide some distance between the gold electrodes and the central benzene ring to prevent the quenching of the interference effects caused by the strong hybridization between the molecular orbitals and the continuous density of states of the electrodes. The thiol anchoring groups, providing a covalent linkage to the electrodes, are the most common choice to form single-molecule junctions. The acetyl protection group is frequently introduced in conjugated molecules to avoid the oxidative polymerization of free thiols. These acetyl groups are cleaved spontaneously at the gold surfaces or upon exposure to an acidic or a basic environment [27,28].

The low-bias conductance and formation of single-molecule junctions were studied using the MCBJ technique. The lithographic MCBJ device consists of a narrow gold constriction patterned on top of a flexible phosphor-bronze substrate coated with a polyimide layer for insulation [29]. The layout of the MCBJ device clamped in a three-point bending configuration is shown in Figure 1b. By driving the pushing rod against the bottom part of the MCBJ device, the gold constriction is stretched until it breaks, leaving a pair of sharp electrodes separated by a nanometer-scale gap. Once the bridge is broken, atomic-sized gold contacts were repeatedly formed and broken by moving the electrodes towards and away from each other at a speed of 9 nm/s. Simultaneously, using a logarithmic amplifier the conductance *G* = *I*/*V* was measured with a bias voltage of 0.1 V applied across the electrodes.

## Results and discussion

**Figure 2:**
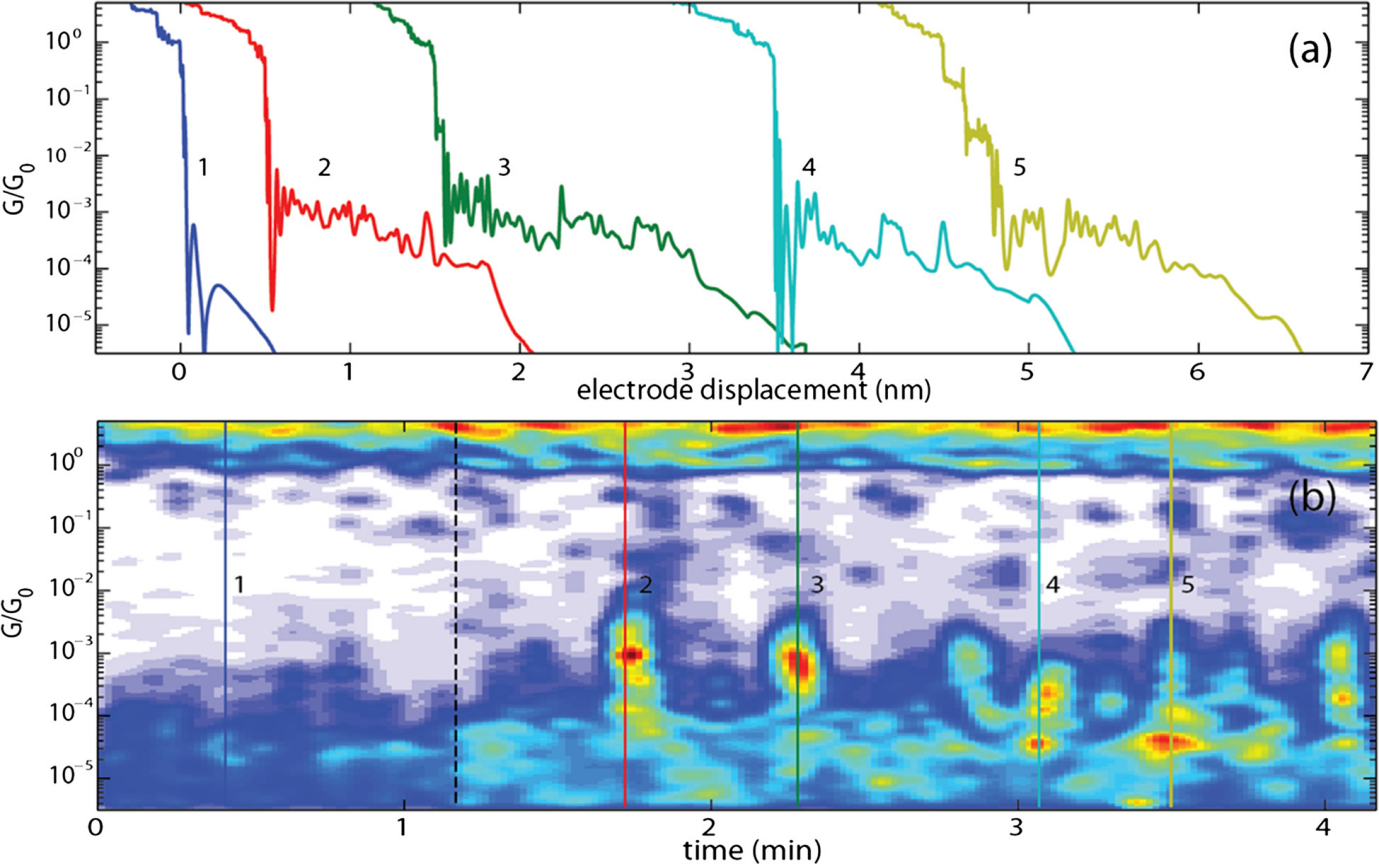
**Formation of molecular junctions, after the deposition of a droplet of 1 mM solution of *para*-OPV3 molecules onto the MCBJ device.** (**a**) Examples of individual breaking traces for junction exposed to (1) 1,2-dichlorobenzene and (2, 3, 4, and 5) 1 mM solution of *para*-OPV3 molecules in 1,2-dichlorobenzene. (**b**) 2D-conductance map while depositing a 2-μL drop of 1 mM solution of *para*-OPV3 molecules in 1,2-dichlorobenzene at around 1 min indicated by the black dashed line.

The molecules were deposited onto the MCBJ device by pipetting a 2-μL droplet of a freshly prepared 1 mM solution in 1,2-dichlorobenzene. In order to exclude artifacts resulting from contaminant species adsorbed on the gold surface, the characterization of the MCBJ device was first performed in pure 1,2-dichlorobenzene. The breaking traces measured in the presence of 1,2-dichlorobenzene (see 1 at Figure 2a) exhibit a flat plateau close to the conductance quantum, *G*_0_(= 2 e^2^/h). This plateau characterizes the formation of a contact consisting of a single Au-Au bond bridging the gap between the electrodes. Upon further stretching, the metallic contact breaks which is observed as an abrupt conductance drop to a value ranging from 10^−3 ^to 10^−4 ^*G*_0_. Beyond this point, electron tunneling between the electrodes leads to an exponential conductance decay with increasing electrode displacement, as expected for tunneling between metal electrodes. The abrupt drop in conductance after the separation of the electrodes is generally observed during the breaking of gold contacts, and it has been associated to the mechanical relaxation and atomic rearrangements at the electrode apexes [30].

The formation of molecular junctions is illustrated in the two-dimensional conductance map in Figure 2b. This 2D-conductance map has been obtained by collecting the conductance histogram in color code of 250 consecutive breaking traces as those shown in Figure 2a. After about 1 min (dashed black line) recording breaking traces for a junction exposed to 1,2-dichlorobenzene, a 2-μL drop of 1 mM solution of *para*-OPV3 molecules is deposited onto the MCBJ device. As shown in Figure 2, the introduction of the molecules produces a notable change on the shape of the breaking traces. The characteristic plateau close to one *G*_0_ remains present, but additional plateau-like structures are now observed at conductance values around 10^−3 ^to 10^−4 ^*G*_0_ (see Figure 2b). The breaking traces measured in the presence of *para*-OPV3 molecules show a predominant occurrence of such plateaus as evidenced in Figure 2b by the yellow/orange regions at these conductance values. These conductance plateaus are the signature of the formation of molecular junctions. We have observed that by adding 2 meq of tetrabutylammonium hydroxide (Bu_4 _NOH) to the solution, the probability of forming such junctions increases. Roughly, we found that the number of traces with plateaus is about two times higher in the presence of this deprotecting agent. We ascribe this observation to the increased reactivity of free thiols to the gold surface with respect to the acetyl-protected thiols.

**Figure 3:**
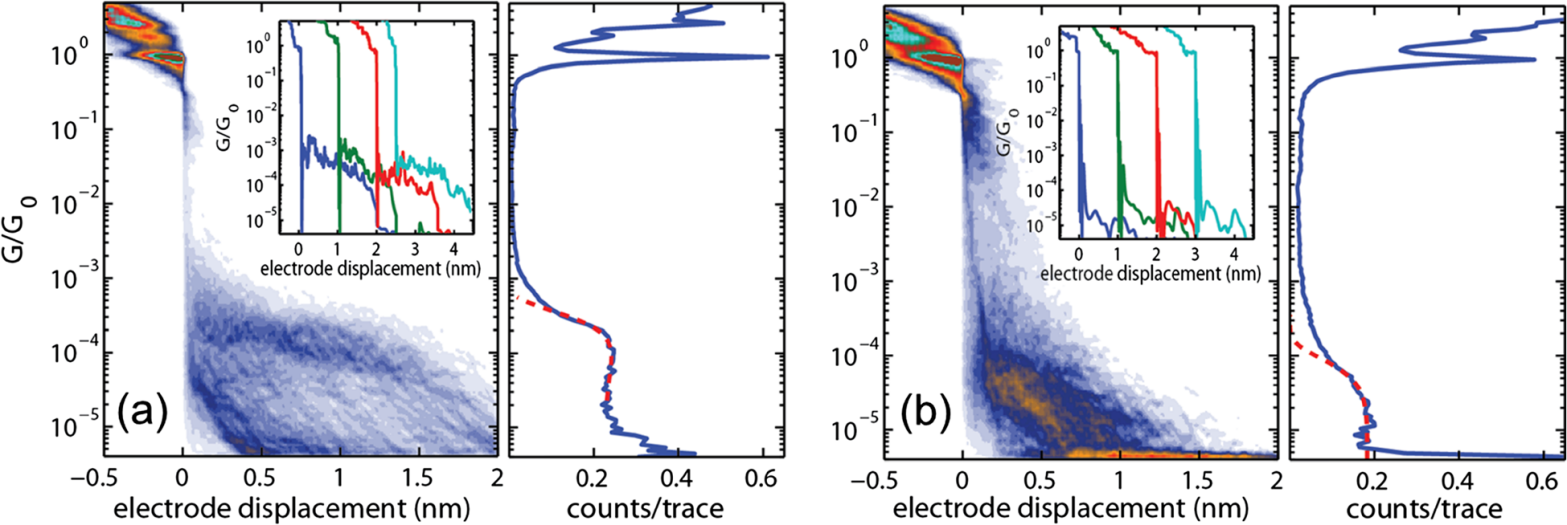
**Two-dimensional trace histogram.** Two-dimensional trace histogram constructed from 1,000 consecutive breaking traces measured at room temperature and 0.1 V bias voltage for MCBJ devices exposed to 1 mM solution of (**a**) *para*-OPV3 and (**b**) *meta*-OPV3 molecules in 1,2-dichlorobenzene. Regions of high counts (blue areas) represent the most probable evolution during the breaking of the contact. The most probable conductance values were extracted by fitting the characteristic peak of the 1D-conductance histograms (right) to a Gaussian function (red dashed curve).

To confirm reproducibility, we have performed several measurements for *para*- and *meta*-OPV3 molecules during different days and using different MCBJ devices. In Figure 3 typical trace histograms [31] and one-dimensional histograms (right panel) built from 1,000 consecutive breaking traces measured in the presence of the molecules are shown. To build the trace histograms, the individual traces (as the ones shown in the inset) were shifted horizontally to fix the rupture of Au-Au contacts at zero electrode displacement. The color scale in the trace histogram indicates the density of data points found at each displacement and conductance value, and, therefore, the colored areas represent the most probable evolution during the breaking process.

The one-dimensional conductance histograms of Figure 3 show broad peaks centered at 1.1 × 10^−4 ^*G*_0_ and 1.5 × 10^−5 ^*G*_0_ for *para*-OPV3 and *meta*-OPV3 molecules, respectively. These values have been obtained from a Gaussian fit (showed as dashed red lines in the 1D conductance histograms). The trace and the 1D conductance histograms show conductance variations around these values. It is well known that the electron transport through a molecule depends on the local environment and the nature of metal/molecule interfaces. They affect the formation and stability of single-molecule junctions, giving rise to variations in the conductance [22].

**Figure 4:**
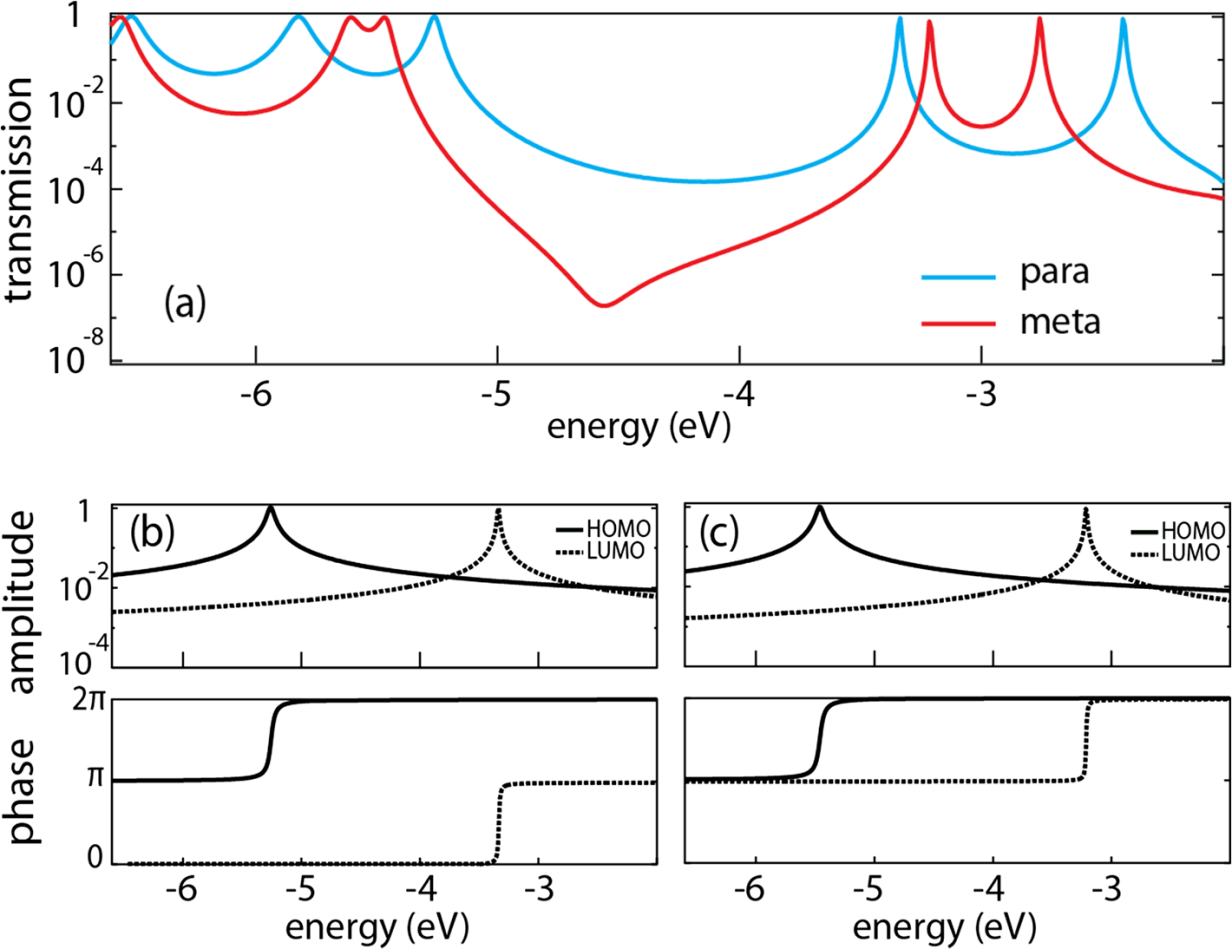
**Calculated transmissions through the π-systems of both molecules.** (**a**) Calculated transmission of *para*- and *meta*-OPV3 derivatives in gas phase. (**b**) Amplitude and phase of the transmission through the HOMO and LUMO of a *para*-OPV3 molecule. (**c**) Amplitude and phase of the transmission through the HOMO and LUMO of a *meta*-OPV3 molecule.

The dramatic suppression in conductance cannot be explained from a single-barrier tunneling mechanism, because the *meta*-OPV3 is shorter than the *para*-OPV3 and therefore should be more conductive. In contrast, the differences in conductance can be understood as a form of quantum interference. In order to get a deeper insight into the interference phenomenon, we have performed non-equilibrium Green’s function calculations using the ground-state electron density (of the molecules in gas phase) obtained from the density functional theory. In Figure 4, the calculated transmissions through the π-systems of both molecules are shown. At energies between the HOMO and LUMO levels, the transmission of the *meta*-OPV3 molecule is more than an order of magnitude smaller than that of a *para*-OPV3, with an anti-resonance occurring at 4.56 eV, where the transmission drops substantially. This drop is caused by the destructive interference between transmission coefficients of different orbitals. In the Landauer formalism, the charge propagation through molecules can be described as a transmission through different molecular orbitals [7]. Using the non-equilibrium Green’s function formalism, it is possible to separate the total transmission into contributions from the individual molecular orbitals. Since these contributions are complex (i.e., they have an amplitude and a phase), interference effects can arise when transmission through different orbitals are combined.

The amplitudes of the transmissions are approximately the same for both molecules, however, the phase of the transmission through the LUMO differs by π from *para*- to *meta*-OPV3, while the phase of the HOMO is the same (see Figure 4b,c). This results in constructive interference for a *para*-OPV3 molecule when the transmission through the HOMO and LUMO are combined. For *meta*-OPV3 molecule, the phase shift results in destructive interference between the HOMO and LUMO transmission, as evident from the drop in the full transmission plot (Figure 4a). It should be noted that also the HOMO-1 and LUMO+1 orbitals contribute to the transmission within the HOMO-LUMO gap. The phase behavior of these orbitals is the same as for the HOMO and LUMO, i.e., constructive and destructive interference for *para*- and *meta*-OPV3 molecules, respectively. Note that the transmission of the *meta*-OPV3 does not go to zero at the anti-resonance due to the contributions from the HOMO-2 and HOMO-3 orbitals. This analysis therefore confirms the occurrence of constructive and destructive interferences in the molecules studied experimentally.

## Conclusion

In conclusion, we have shown that the low-bias conductance through a single *meta*-OPV3 molecule is one order of magnitude smaller that through a *para*-OPV3 one. Non-equilibrium Green’s function calculations show that the difference in conductance between *para*- and *meta*-OPV3 can be explained by the occurrence of interference effects in the central benzene ring. The results open up new possibilities for the design of single-molecule devices based on quantum interference effects, for instance, switching devices that operate by combining destructive and constructive molecular structures.

## Competing interests

All the authors declare no competing interests.

## Authors’ contributions

The experiments, including the analysis of data, were conceived and performed by CA and RF. HvdZ also conceived and co-wrote the paper. The synthesis of the molecules was done by KM and TB, and the calculations were performed by JS. All authors read and approved the final manuscript.

## Supplementary Material

Additional file 1 **Supporting information.** Discussion of synthesis of *meta*-OPV3 and its experimental details. Click here for file
