# Supplementary material for: Quantum interference effects at room temperature in OPV-based single-molecule junctions
Source: Nanoscale Res Lett. 2013 May 16;8(1):234. doi: 10.1186/1556-276X-8-234 (PMC3663707; doi:10.1186/1556-276X-8-234)
Supplement: Additional file 1 — Supporting information. Discussion of synthesis of meta-OPV3 and its experimental details. [file 1556-276X-8-234-S1.docx]

**Quantum interference effects at room temperature in OPV based single-molecule junctions**

**Supporting Information**

Carlos R. Arroyo,^1,*^ Riccardo Frisenda,^1^ Kasper Moth-Poulsen,^2,^† Johannes S. Seldenthuis,^1^ Thomas Bjørnholm,^2^ Herre S.J. van der Zant^1,*^

*1. Kavli Institute of Nanoscience, Delft University of Technology, Lorentzweg 1, 2628 CJ*

*Delft, The Netherlands*

*2. Nano-Science Center & Department of Chemistry, University of Copenhagen,Universitetsparken 5, DK-2100 Copenhagen, Denmark*

* E-mail: [c.arroyorodriguez@tudelft.nl](mailto:c.arroyorodriguez@tudelft.nl); h.s.j.vanderzant@tudelft.nl

† Present address: *Dept. Chemical & Biological Eng., Chalmers University of Technology, 41296 Gothenburg, Sweden*

**Synthesis of *meta*-OPV3:**

*Meta*-OPV3 was synthesized via our previously described Horner-Wadsworth-Emmons strategy [[^1^](#_ENREF_1)]. The thiol was protected as a *tert.* butyl thioether throughout the synthetic sequence and converted into a base labile thioester in the last reaction step [[^2^](#_ENREF_2)].

******

**Figure S1.** Synthesis of *meta*-OPV3.

**Experimental Details (synthesis).**

^1^ H NMR (300 MHz) and ^13^ C NMR (75 MHz) spectra were recorded on a Varian instrument.

Samples were prepared using CDCl_3_ purchased from Cambridge Isotope Labs. Electron impact ionization mass spectrometry (EI-MS) was performed on a Varian MAT 311A. Microanalyses were performed at the Microanalytical Laboratory at the Department of Chemistry, University of Copenhagen. All chemicals obtained from Aldrich, except for 4-(tert-butylthio)benzylphosphonate that were prepared according to reported procedures [[^1^](#_ENREF_1)].

***Trans*-1,3-Bis(4-tert-butylthiostyryl)benzene**

Isophtalaldehyde (671 mg, 5 mmol) and 4-(tert-butylthio)benzylphosphonate (3.16g, 10 mmol) was dissolved in tertrahydrofuran (80 mL). Potassium tert. Butylate (1.23 g, 11 mmol) was added slowly in small portions, the reaction mixture was stirred at room temperature for 1½ hours and quenched in ice/water. Extraction with toluene (4 times 60 mL) drying (MgSO_4_) and evaporation of the solvent in vacuo afforded the crude product, which was recrystallised by refluxing in toluene (10 mL) containing catalytic amounts of iodine. The product crystallized as white needles by leaving the mixture at -16 °C over night, m = 1.88 g, corresponding to 82% yield.

MP: 144.6-145.9 °C, MS (FAB, m-NBA) shows a peak at 458.2 (M^+^) H NMR (250 MHz, CDCl_3_): δ 1.25 (s, 18H), 7.09 (s, 4H), 7.26-7.36 (m, 3H), 7.42 (d, J=8.6Hz, 4H), 7.48 (d, J=8.6Hz, 4H), 7.58 (broad singlet, 4H) ^13^C (62.9 MHz, CDCl_3_) δ 29.99, 45.24, 123.95, 125.02, 125.49, 127.33, 128.10, 128.36, 131.13, 136.56 (2 peaks) 136.76.

***Trans*-1,3-Bis(4-acetylthiostyryl)benzene (*meta*-OPV3)**

*Trans*-1,3-Bis(S-tert.butylthiostyryl)benzene (0.91 g, 2 mmol), was dissolved in a mixture of dichloromethane (20 mL) and toluene (40 mL). Acetyl chloride (1 mL) was added, and the reaction mixture cooled to 0°C. Borontribromide (5 mL, 1.0 M solution in dichloromethane) was added during a period of 15 min, and the reaction mixture stirred for 45 min with cooling. The reaction mixture was stirred for further 11h at room temperature. The reaction mixture was quenched on ice, extraction with dichloromethane (4 times 100 mL), drying (MgSO_4_) and evaporation of the solvent in vacuo afforded a crude material which was purified by column chromatography to yield 400 mg of 1,3-Bis(S-acetylthiostyryl)benzene corresponding to 47%, recrystallisation by refluxing for 12h in toluene containing catalytic amounts of iodine afforded the *Trans*-1,3-Bis(S-acetylthiostyryl)benzene as white needles, m = 143 mg corresponding to 17% yield. MP: 168.8-170.8 °C Anal. Calculated for C_26_H_22_S_2_O_2_: C, 72.52; H, 5.15; S, 14.89. Found: C, 72.19; H, 5.11; S, 14.87. MS (EI) 430 (90), 346 (100). H NMR (400 MHz, CDCl_3_): δ 2.38 (s, 6H), 7.07 (d, J=16 Hz, 2H), 7.12 (d, J=16Hz, 2H), 7.28-7.39 (several multiplets, 7H), 7.49 (d, J=8.7Hz, 4 H), 7.58 (t, J=1Hz, 1H) ^13^C (100.57 MHz, CDCl_3_) δ 30.12, 124.93, 126.09, 128.82, 127.08, 128.00, 128.99, 129.79, 134.58, 137.29, 138.33, 193.92.

**References:**

1. Stuhr-Hansen N, Christensen JB, Harrit N, Bjørnholm T: **Novel synthesis of protected thiol end-capped stilbenes and oligo (phenylenevinylene) s (OPVs)**. *The Journal of Organic Chemistry* 2003, **68**: 1275-1282.

2. Stuhr-Hansen N: **The tert-Butyl Moiety—A Base Resistent Thiol Protecting Group Smoothly Replaced by the Labile Acetyl Moiety**. *Synthetic Communications* 2003, **33**: 641-646.
